# Immortalized Parkinson's disease lymphocytes have enhanced mitochondrial respiratory activity

**DOI:** 10.1242/dmm.025684

**Published:** 2016-11-01

**Authors:** Sarah J. Annesley, Sui T. Lay, Shawn W. De Piazza, Oana Sanislav, Eleanor Hammersley, Claire Y. Allan, Lisa M. Francione, Minh Q. Bui, Zhi-Ping Chen, Kevin R. W. Ngoei, Flora Tassone, Bruce E. Kemp, Elsdon Storey, Andrew Evans, Danuta Z. Loesch, Paul R. Fisher

**Affiliations:** 1 Discipline of Microbiology, Department of Physiology Anatomy and Microbiology, School of Life Sciences, College of Science Health and Engineering, La Trobe University, Melbourne, Victoria 3086, Australia; 2 Department of Psychology and Counselling, School of Psychology and Public Health, College of Science Health and Engineering, La Trobe University, Melbourne, Victoria 3986, Australia; 3 Centre for Epidemiology and Biostatistics, Melbourne School of Population and Global Health, University of Melbourne, Melbourne, Victoria 3010, Australia; 4 Department of Medicine, University of Melbourne St. Vincent's Institute of Medical Research, Fitzroy, Victoria 3065, Australia; 5 UC Davis MIND Institute, Sacramento, CA 95817, USA; 6 Department of Medicine (Neuroscience), Monash University, (Alfred Hospital Campus), Commercial Road, Melbourne, Victoria 3004, Australia; 7 Department of Neurology, Royal Melbourne Hospital, Parkville, Victoria 3052, Australia

**Keywords:** Parkinson's disease, Lymphoblast, Lymphocyte, Mitochondria, Respiration, AMPK, Complex I, ATP, OXPHOS, ROS, Oxidative stress

## Abstract

In combination with studies of post-mortem Parkinson's disease (PD) brains, pharmacological and genetic models of PD have suggested that two fundamental interacting cellular processes are impaired – proteostasis and mitochondrial respiration. We have re-examined the role of mitochondrial dysfunction in lymphoblasts isolated from individuals with idiopathic PD and an age-matched control group. As previously reported for various PD cell types, the production of reactive oxygen species (ROS) by PD lymphoblasts was significantly elevated. However, this was not due to an impairment of mitochondrial respiration, as is often assumed. Instead, basal mitochondrial respiration and ATP synthesis are dramatically elevated in PD lymphoblasts. The mitochondrial mass, genome copy number and membrane potential were unaltered, but the expression of indicative respiratory complex proteins was also elevated. This explains the increased oxygen consumption rates by each of the respiratory complexes in experimentally uncoupled mitochondria of iPD cells. However, it was not attributable to increased activity of the stress- and energy-sensing protein kinase AMPK, a regulator of mitochondrial biogenesis and activity. The respiratory differences between iPD and control cells were sufficiently dramatic as to provide a potentially sensitive and reliable biomarker of the disease state, unaffected by disease duration (time since diagnosis) or clinical severity. Lymphoblasts from control and PD individuals thus occupy two distinct, quasi-stable steady states; a ‘normal’ and a ‘hyperactive’ state characterized by two different metabolic rates. The apparent stability of the ‘hyperactive’ state in patient-derived lymphoblasts in the face of patient ageing, ongoing disease and mounting disease severity suggests an early, permanent switch to an alternative metabolic steady state. With its associated, elevated ROS production, the ‘hyperactive’ state might not cause pathology to cells that are rapidly turned over, but brain cells might accumulate long-term damage leading ultimately to neurodegeneration and the loss of mitochondrial function observed post-mortem. Whether the ‘hyperactive’ state in lymphoblasts is a biomarker specifically of PD or more generally of neurodegenerative disease remains to be determined.

## Abstract (precis)

**Editors' choice:** Cultured Parkinson's disease lymphocytes are metabolically hyperactive, suggesting a new understanding of the underlying cytopathology and biomarkers for this and potentially other neurodegenerative diseases.

## INTRODUCTION

Two major, interconnected pathways have been implicated as producing cytopathology in idiopathic Parkinson's disease (iPD). The first pathway is related to protein misfolding and aggregation either as the result of increased aggregate production or decreased elimination (Schapira et al., 2009; Schapira, 2011). The second pathway is related to mitochondrial dysfunction, especially of the mitochondrial respiratory chain largely involving mitochondrial complex I (Schapira and Gegg, 2011; Keane et al., 2011; Exner et al., 2012; Perier and Vila, 2012). However, currently there is not a clear understanding of the link between cellular toxicity, mitochondrial changes and the disease process. Previous reports on mitochondrial dysfunction and PD have resulted in a commonly held view that reductions in mitochondrial respiratory function contribute to PD cytopathology (Schapira and Gegg, 2011; Keane et al., 2011; Exner et al., 2012; Perier and Vila, 2012). The evidence can be summarized as follows:

(1) Mitochondrial defects directly or indirectly affecting complex I, caused either by mutations affecting mitochondrial proteins or by certain toxins [e.g. rotenone, 1-methyl-4-phenyl-1,2,3,6-tetrahydroperidine (MPTP), paraquat], can cause PD in humans and animal models. However, this does not prove that impaired mitochondrial function plays a causative role in the majority of PD cases, which are idiopathic.

(2) Post-mortem brains of individuals with PD exhibit reduced mitochondrial complex I activity and reduced expression of many genes encoding mitochondrial proteins. However, it is not clear whether this reduced mitochondrial function in post-mortem PD brains is a cause or a result of the disease process.

(3) Elevated production of reactive oxygen species (ROS; presumably derived from leakage of electrons directly to molecular oxygen at the point where they would normally pass from Complex I or II via ubiquinone to Complex III) has been consistently reported from various cell types in individuals with PD. However, elevated ROS production can result either from impaired mitochondrial electron transport or from increased rates of otherwise normal mitochondrial respiration.

Direct assays of mitochondrial respiratory function or enzyme activity in various cell types derived from small numbers of living individuals with idiopathic PD (iPD) have produced contradictory and collectively inconclusive results (Bravi et al., 1992; Yoshino et al., 1992; Barroso et al., 1993; Martín et al., 1996; Parker and Swerdlow, 1998; Müftüoğlu et al., 2003; Shinde and Pasupathy, 2006; Esteves et al., 2010; Cronin-Furman et al., 2013; Ambrosi et al., 2014). To pursue this issue, we studied multiple parameters of mitochondrial function in lymphoblastoid cells (hereafter referred to as lymphoblasts) derived from peripheral blood lymphocytes of individuals with iPD and age-matched controls. Why did we choose blood-derived cells when, by definition, the pathological process in iPD involves neural tissue in the brain? Firstly, blood is a highly accessible human tissue, unlike the brain. Accordingly, there is a great need for blood biomarkers of PD disease and progression, yet this has been little studied. Secondly, recent evidence shows clearly that blood cells are also somehow embroiled in the pathology of iPD. Thus, several studies have revealed genes whose expression is altered in iPD blood cells (Scherzer et al., 2007; Molochnikov et al., 2012; Karlsson et al., 2013; Soreq et al., 2013; Zhang et al., 2014), some of them already known to exhibit altered expression levels in post-mortem *substantia nigra* of individuals with iPD (Grünblatt et al., 2004; Simunovic et al., 2009; Mandel et al., 2005). These include ALDH1A1 (aldehyde dehydrogenase family H1 subfamily A1, also known as retinal dehydrogenase 1), PSMC4 (26S protease regulatory subunit 6B) and SKP1A (S-phase kinase-associated protein 1A), all of which exhibited reduced transcript levels in PD, and HSPA8 (heat shock 70 kDa protein 8, also known as heat shock cognate 71 kDa protein) whose transcript levels are elevated in PD (Molochnikov et al., 2012).

The implication is that the cytopathology of iPD extends to blood cells and that the differences between iPD and control lymphoblasts might not only shed light on the underlying disease processes but also provide readily accessible biomarkers for disease and/or its progression. We report here that immortalized lymphocytes from individuals with iPD and healthy controls do indeed exhibit remarkable metabolic differences in the form of a dramatic elevation of mitochondrial respiratory activity in iPD cells. This is accompanied by a concomitant increase in the production of ROS, a cytotoxic byproduct of respiration.

## RESULTS

### ROS production is elevated in iPD lymphoblasts, but mitochondrial membrane potential is unaltered and ATP steady-state levels are increased

**Fig. 1.**
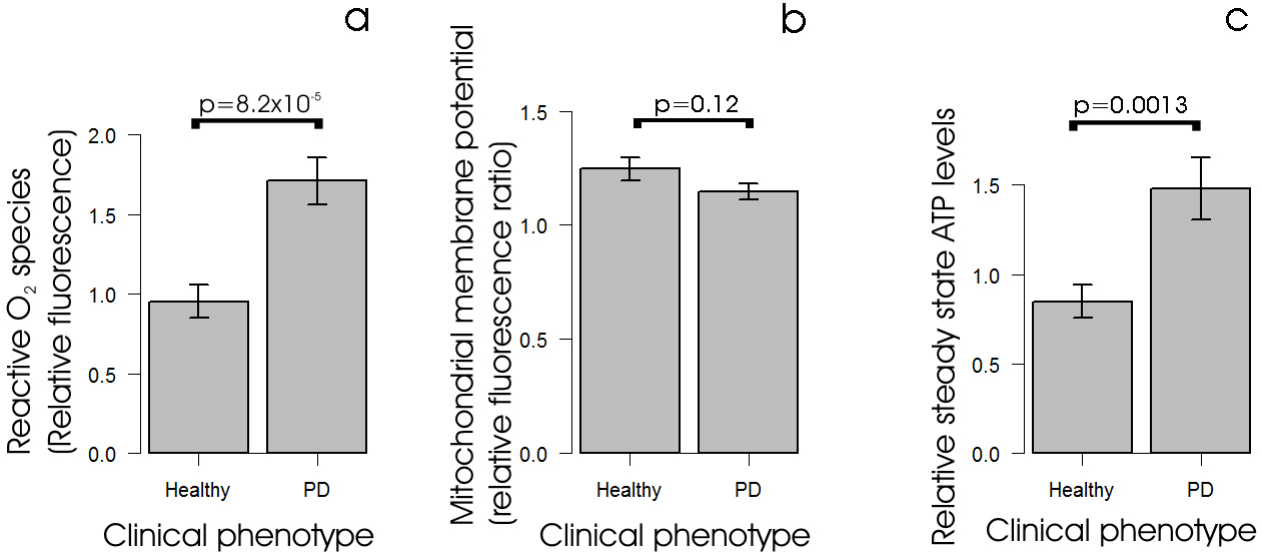
**Alterations to parameters of mitochondrial function in PD lymphoblasts.** (A) Reactive O_2_ species levels are elevated in lymphoblasts from individuals with PD. Intracellular ROS levels were measured in lymphoblasts from PD and control individuals using MAK142 (Deep Red) fluorescence. Except for one control cell line (which was assayed only once), the mean normalized fluorescence from 10^5^ cells was measured in duplicate in at least three independent experiments. The ROS fluorescence in the PD lines (*n*=30) was significantly elevated compared with controls (*n*=9) (single-sided Welch test). (B) Mitochondrial membrane potential is unaltered in iPD lymphoblasts. The relative mitochondrial membrane potential (Δψ_m_) was measured in lymphoblasts from PD and control individuals using the ratio of MitoTracker Red CMXRos (Δψ_m_-dependent) to MitoTracker Green (mitochondrial mass-dependent) fluorescence. Each PD (*n*=30) and control (*n*=9) cell line was assayed in duplicate in at least three independent experiments and means were calculated. The mitochondrial membrane potential was not significantly different in the PD and control samples (two-sided Welch *t*-test). (C) Steady-state ATP levels are elevated in lymphoblasts from individuals with PD. Steady-state ATP levels were assayed using a luciferase-based luminescence assay in lymphoblasts from PD and control individuals. Each PD (*n*=30) and control (*n*=9) cell line was assayed in duplicate in at least three independent experiments and means were calculated. The steady-state ATP levels in the PD lines were elevated significantly (single-sided Welch test). Error bars are standard errors of the mean (s.e.m.).

Previous work has shown that cells from various tissues exhibit elevated ROS production in individuals with PD compared with controls (Dias et al., 2013). We therefore measured ROS production in lymphoblasts from individuals with iPD and controls and found, as expected, that ROS production was significantly elevated in the cells from individuals with iPD compared with those from an age-matched control group (Fig. 1A). This elevation of ROS production could be caused by a blockade of the normal electron flow from complex I and II through complexes III and IV to molecular oxygen, leading to increased diversion of electrons directly to molecular oxygen. Indeed, it is typically interpreted in this way. If the elevated ROS production in iPD lymphoblasts was caused by a blockade of electron transport at or downstream of the transfer of electrons to complex III and IV, it should be accompanied by a reduction in mitochondrial membrane potential. When we measured this, however, we found no significant reduction in mitochondrial membrane potential in iPD lymphoblasts compared with controls (Fig. 1B). Another possible explanation for elevated ROS production is that it simply results from increased rates of electron transport, accompanied by an associated increase in the rate of leakage of electrons directly to molecular oxygen. If this is true then the elevated ROS production in iPD cells should be accompanied by increased mitochondrial ATP production, whereas the reverse should be true if the production of ROS is caused by a partial blockade of normal respiratory electron transport. Fig. 1C shows that iPD lymphoblasts exhibited elevated steady-state levels of ATP compared with control lymphoblasts, supporting the idea that in the iPD cells ATP production is increased.

### Mitochondrial respiratory complexes are functionally normal but dramatically more active in iPD lymphoblasts

**Fig. 2.**
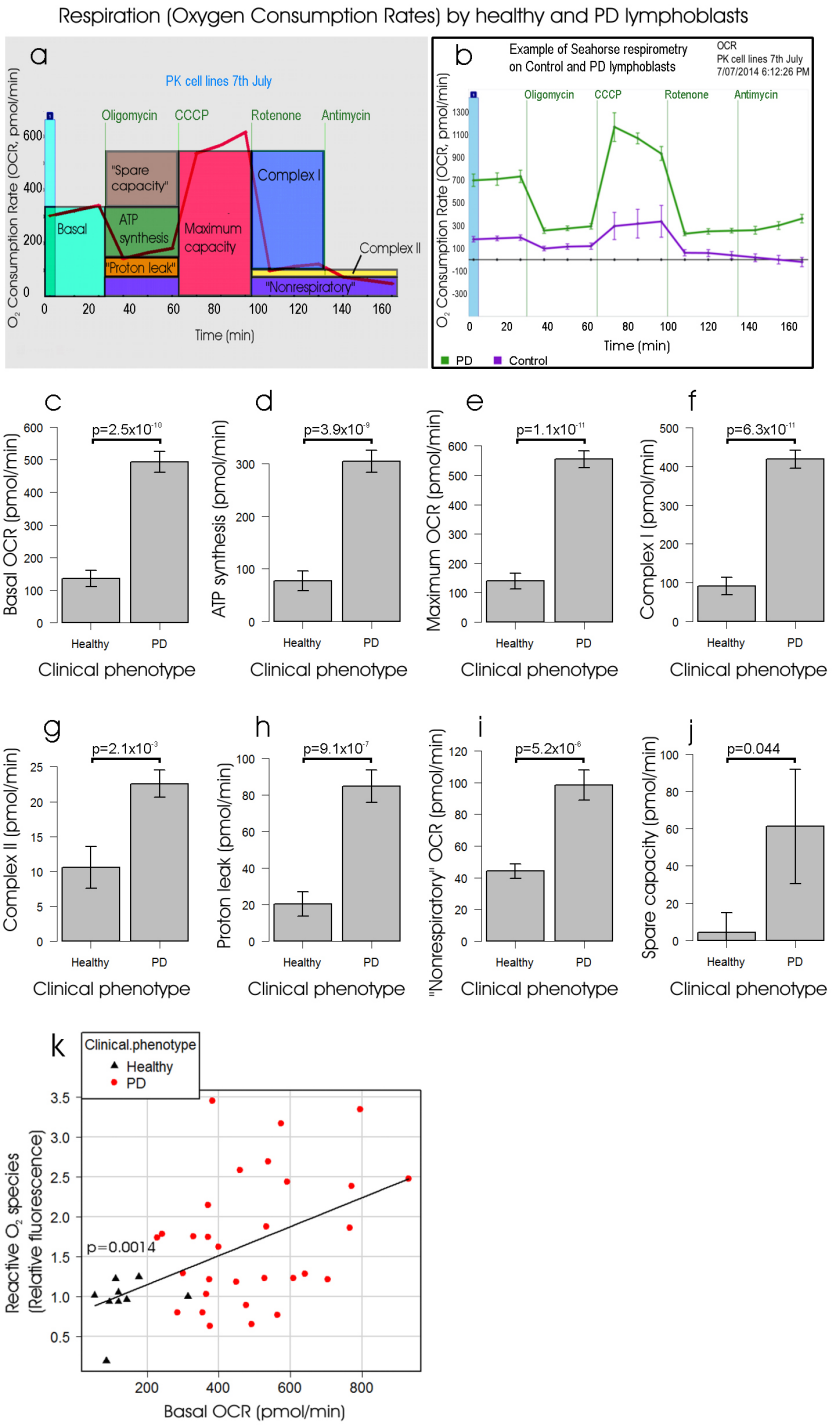
**The rate of O_2_ consumption by mitochondrial respiration is elevated in lymphoblasts** **from individuals with PD.** Oxygen consumption rates (OCR in pmol/min) were measured using a Seahorse Extracellular Flux Analyzer for 8×10^5^ lymphoblasts from PD and control individuals. In each experiment the OCR was measured before (basal respiration) and after successive addition of oligomycin (ATP synthase inhibitor), CCCP (uncoupling protonophore), rotenone (complex I inhibitor) and antimycin A (complex III inhibitor), allowing determination of each of the components of respiration as shown in panel (A). In each experiment, data was collected and averaged from four separate wells for each individual cell line. A typical example of an experiment with standard errors reflecting the between-well variance within the experiment is shown in panel (B). Each PD (*n*=30) and control (*n*=9) cell line was assayed in at least three independent experiments and means were calculated. From this data, we determined the OCR attributable to (C) basal respiration, (D) ATP synthesis, (E) uncoupled (maximum) respiration, (F) complex I activity, (G) complex II activity, (H) mitochondrial activities other than ATP synthesis (‘proton leak’), (I) processes not driven by electron transport (‘non-mitochondrial’) and (J) the spare capacity (excess of uncoupled over basal respiration). Significance probabilities are shown for each of the respiratory parameters. All were elevated significantly in PD lymphoblasts compared with controls (*P*<0.05, single-sided Welch *t*-test). Error bars are s.e.m. (K) Multiple regression analysis of the relationship between ROS production and the parameters of respiration shown in panels C to J showed that ROS levels were correlated with the rate of basal respiration. The analysis incorporated dummy variables to allow distinction between control and PD lymphoblasts and successive removal of least significant coefficients until only significant coefficients remained. The only remaining significant coefficient (apart from the intercept) was that shown, relating ROS levels to basal metabolic rate, so that both control and PD values lay on the same regression line. The significance of the final regression is shown (*P*<0.05, two-sided *t*-test).

An alternative to the conclusion that ATP production is increased in iPD cells is that mitochondrial ATP production rates were actually lower in the iPD cells, but that ATP consumption rates were also reduced, such that steady-state ATP levels increased. To distinguish these possibilities, we used respirometry with a Seahorse XF^e^24 Extracellular Flux Analyser (Fig. 2A,B) to determine if the flux of electrons from complexes I or II via complexes III and IV to molecular oxygen was elevated or reduced in iPD lymphoblasts compared with controls. Oxygen consumption rates (OCR in pmol/min) were measured before (basal OCR, Fig. 2C) and after successive addition of oligomycin (ATP synthase inhibitor), CCCP (carbonyl cyanide m-chlorophenyl hydrazone, an uncoupling protonophore), rotenone (complex I inhibitor) and antimycin A (complex III inhibitor). From the resulting data we determined the oligomycin-sensitive OCR associated with respiratory ATP synthesis (Fig. 2D), the maximum OCR in uncoupled mitochondria (Fig. 2E) and the OCR attributable to complex I activity (Fig. 2F), complex II activity (Fig. 2G), mitochondrial functions (e.g. protein import) that are not ATP-driven (so-called ‘proton leak’, Fig. 2H), non-respiratory oxygen consumption (e.g. by cellular and mitochondrial oxygenases and oxidases, Fig. 2I), and the respiratory ‘spare-capacity’ (excess capacity of the respiratory electron transport chain that is not being used in basal respiration, Fig. 2J). The results showed that in iPD lymphoblasts, mitochondrial respiration and ATP synthesis rates are dramatically elevated. This suggests that ROS production is higher in individuals with PD because mitochondrial electron transport rates are elevated. To verify if this is so, we conducted a multiple regression analysis relating ROS levels to all of the above respiratory parameters. ROS levels were significantly correlated with basal metabolic rate (*P*=0.0014) and none of the other variables had any significant additional effect on ROS levels (Fig. 2K). Furthermore, there was no difference in this regression relationship between control and PD cells. This result shows that ROS levels are elevated in PD lymphoblasts because of elevated respiration rates, not because of an impairment or blockade of mitochondrial electron transport in the electron transport chain.

**Fig. 3.**
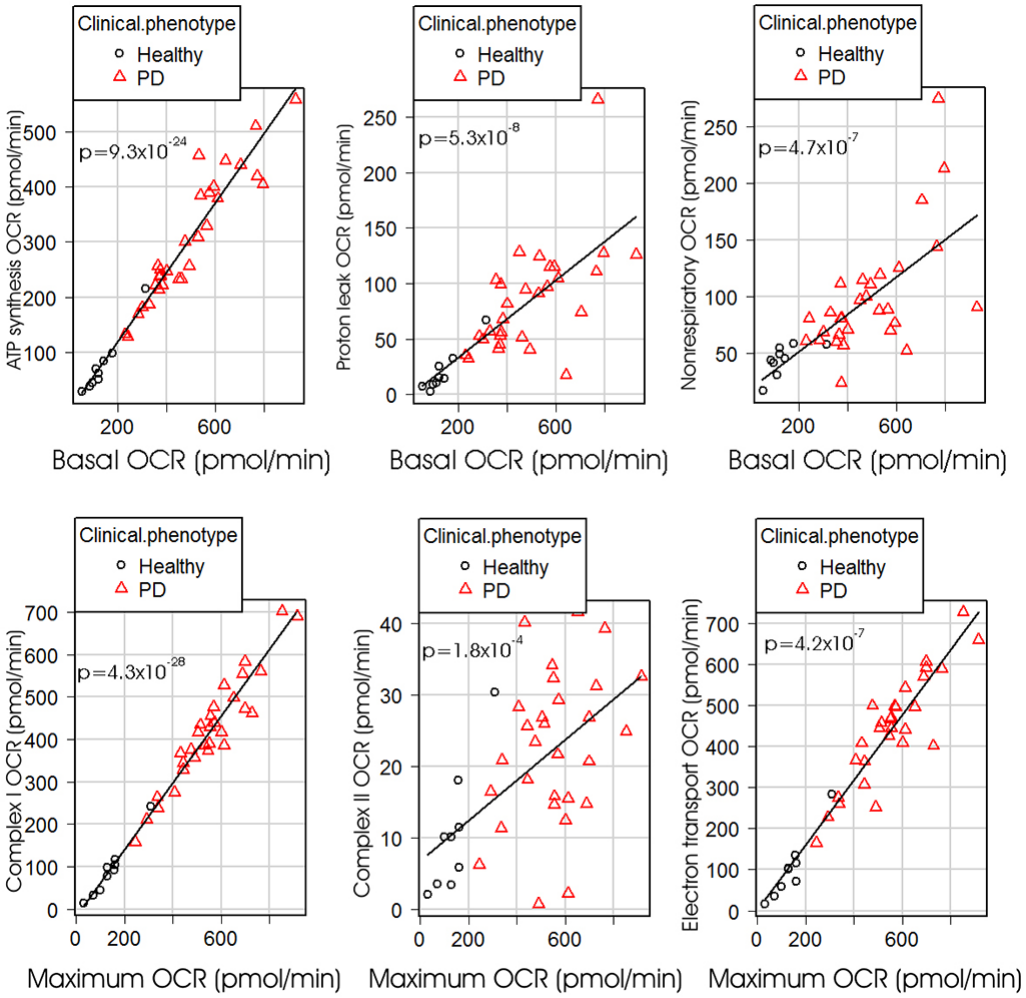
**Regression relationships amongst the components of basal and maximum (uncoupled) respiration.** Multiple regression analysis was conducted for each of the illustrated respiratory parameters against either the basal respiration rate (top row) or the maximum (uncoupled) respiration rate and dummy variables distinguishing PD and control groups (bottom row). Least significant variables were removed successively until only significant regression coefficients (*P*<0.05) remained in the model. All regressions were highly significant, but there were no significant differences in the regressions (slope or intercept) between PD and control groups. Plotted regression lines were fitted by the least squares method. Each point represents the mean result from at least three independent experiments on lymphoblasts from a single participant. OCR=O_2_ consumption rate. Electron transport OCR is the sum of the contributions of complex I and complex II to the maximum OCR.

To confirm that mitochondrial electron transport is more active, but unimpaired, in PD lymphoblasts compared with controls, we examined the regression between basal metabolic rate and its electron transport-driven components – O_2_ consumption by ATP synthesis and the ‘proton leak’. These tight regression relationships were also identical for PD and control lymphoblasts (Fig. 3), as were the relationships between the maximum OCR and its component complex I and complex II activities in uncoupled mitochondria (Fig. 3). If specific mitochondrial electron transport complexes had been impaired in the PD lymphoblasts, these regression relationships would have been altered compared with controls; e.g. if complex I activity was impaired it would have made a proportionately smaller contribution to the maximum OCR in PD cells and the slope of the regression relationship would have been reduced. Overall, our data provide compelling evidence that mitochondrial respiratory function is not impaired in PD lymphoblasts.

### Expression of mitochondrial OXPHOS complexes is elevated in iPD lymphoblasts, but AMPK activity and mitochondrial mass are not

**Fig. 4.**
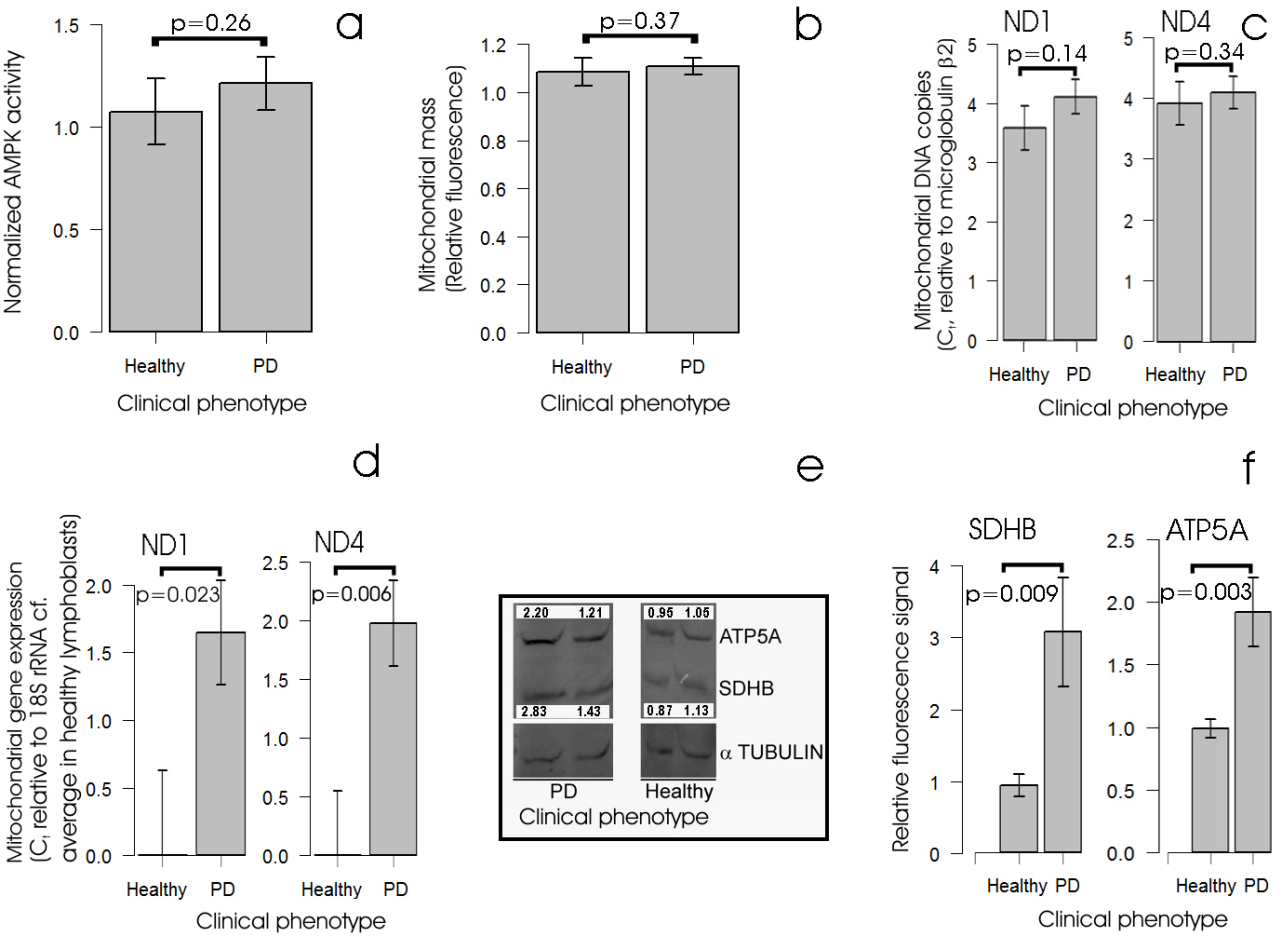
**Steady-state AMPK activity, mitochondrial mass and genome copy number are unchanged in PD lymphoblasts but oxphos mRNA and protein expression are elevated.** (A) AMPK activity is not altered in PD lymphoblasts. Each PD (*n*=30) and control (*n*=8) cell line was assayed in duplicate in each of at least two independent experiments and means were calculated. AMPK activities in the PD cells were not elevated significantly (single-sided Welch *t*-test). Quantitative western blots also revealed no significant change in the ratio of activated AMPK (phosphorylated α1 subunit) to total AMPK in PD cells (Fig. S1A). (B) Mitochondrial mass is not elevated in PD lymphoblasts. Mitochondrial mass was measured in lymphoblasts from PD and control individuals using MitoTracker Green fluorescence. Each PD (*n*=30) and control (*n*=9) cell line was assayed in at least three independent experiments and means were calculated. The fluorescence in the PD lines was not significantly elevated (single-sided Welch test). (C) Mitochondrial genome copy number is unchanged in PD lymphoblasts. Relative mitochondrial genome copy number was measured in duplicate in semi-quantitative RT-PCR as the difference in qPCR threshold cycle number between the mitochondrial genes encoding ND1 and ND4 (mitochondrially encoded subunits of complex I) and the nuclear gene encoding β2-microglobulin. Each PD (*n*=30) and control (*n*=8) cell line was assayed in two or three independent experiments and means were calculated. The mitochondrial genome number was not significantly different in the PD and control cell lines (single-sided Welch tests). Very similar results were obtained using the nuclear 18S rDNA genes as the genome loading control (not shown). (D) Mitochondrial gene expression is elevated in PD lymphoblasts. Quantitative reverse transcription PCR was used to assay ND1 and ND4 mRNAs (mitochondrially encoded subunits of complex I) relative to the nuclear-encoded cytoplasmic 18S rRNA (which provided the internal loading control). Each PD (*n*=26) and control (*n*=7) cell line was assayed in duplicate in one to five independent experiments and mean threshold cycle numbers (C_t_) were calculated. For all cell lines the differences between mRNA expression levels for both subunits and the corresponding averages for the control cell lines were determined. These were found to be elevated significantly in the PD cells (single-sided Welch test). Similar results were found in a subset of samples using the β2-microglobulin mRNA as the internal control (not shown). (E,F) Steady-state levels of OXPHOS proteins are elevated in PD lymphoblasts. (E) Crude lymphoblast protein extracts were separated on SDS-PAGE and western blotted using commercial antibodies. The blot shown is an example with fluorescence signals as shown for the ATP5A and SDHB bands, normalized to the corresponding α-tubulin signal in the same track and expressed relative to the average control values. (F) Background-subtracted fluorescence signals for each band were normalized against the background-subtracted α-tubulin signal in the same track and expressed relative to the average control value. The ATP5A and SDHB levels were significantly higher in PD cells (*n*=11 PD and *n*=6 controls, single-sided Welch test). Similar results were found in experiments with 19 PD samples and four control samples using an anti-β-actin antibody to detect actin as the internal loading control (Fig. S1B,C,D). Error bars are s.e.m.

Considering the foregoing data, we hypothesized that rather than a disease-causing impairment of mitochondrial respiration, PD lymphoblasts exhibit an elevation of mitochondrial activity and turnover that is elicited by a destructive cycle of cellular stress signalling. Under this hypothesis, idiopathic PD cytopathology in these cells would be initiated by a non-mitochondrial dysfunction causing the activation of intracellular stress signalling pathways. Once mitochondrial activities have been elevated in response to stresses such as the accumulation of unfolded proteins, they would in turn cause oxidative stress in the form of increased production of reactive oxygen species. Various PD cell types are known to be subjected to a variety of cellular stresses (Schapira et al., 2009; Schapira, 2011; Dias et al., 2013; Placido et al., 2015). These include oxidative stress and the accumulation of unfolded protein aggregates such as the diagnostic α-synuclein-containing Lewy bodies found in dopaminergic neurons in the *substantia nigra*. PD leukocytes contain elevated levels of the oxidative DNA base damage product 8-OH-2-deoxyguanosine (8-OHDG) (Chen et al., 2009). Furthermore, the levels or activities of a variety of cellular stress response proteins are altered in PD cells or experimental PD models, including molecular chaperones (heat shock proteins) and AMPK (Grünblatt et al., 2004; Simunovic et al., 2009; Mandel et al., 2005; Liu and Chern, 2015; Santini et al., 2015). AMPK is a stress-sensing protein kinase, best known for its activation by energy stress such as occurs as a result of mitochondrial dysfunction (Bokko et al., 2007), but also activated by a variety of other stresses including the accumulation of unfolded protein aggregates, hypoxia, elevated cytoplasmic Ca^2+^ levels and oxidative stress (elevated ROS levels) (Hardie et al., 2012). If the lymphoblasts from individuals with PD are chronically exposed to one or more of the foregoing cellular stresses, then AMPK activity within them could be elevated compared with control lymphoblasts. One of the downstream consequences of elevated AMPK activity in cells is known to be an increase in both the activity and biogenesis of mitochondria (Hardie et al., 2012). This could explain why mitochondrial function is so elevated in iPD lymphoblasts. Conversely, the elevated steady-state ATP levels in iPD lymphoblasts would be expected to inhibit AMPK activity. It was therefore apposite to determine if steady-state AMPK activities are elevated or suppressed in PD lymphoblasts. Fig. 4A shows that AMPK activities are not significantly altered in lymphoblasts from individuals with idiopathic PD. Thus, the dramatic elevation of mitochondrial activity in these cells cannot be ascribed to a constitutively high level of AMPK activity.

Although AMPK is a key upstream regulator of mitochondrial biogenesis and proliferation, it is not the only one (Jornayvaz and Shulman, 2010). It was therefore possible that the increased mitochondrial respiratory activity of these cells were a result of increased mitochondrial mass, even though AMPK activities were unchanged. To determine this, we measured mitochondrial mass and mitochondrial gene copy number in iPD and control lymphoblasts. Neither was significantly increased (Fig. 4B,C), consistent with the unchanged AMPK activities in these cells. We conclude that the increased mitochondrial respiration rates in iPD cells are due to elevated activity of the mitochondria and not to increases in the steady-state mitochondrial mass or genome copy number.

As the same mitochondrial mass in PD lymphoblasts is more active in ATP generation compared with controls, we hypothesized that mitochondrial proteins involved in oxidative phosphorylation (OXPHOS) would be more highly expressed in PD cells compared with controls. To test this, we measured the expression levels of two indicative mitochondrial mRNAs encoded by mitochondrial genes (Fig. 4D) and the levels of two OXPHOS proteins encoded by nuclear genes (Fig. 4E,F) and found all to be significantly greater in PD cells compared with controls. Thus, the high rates of OXPHOS activity in PD lymphoblasts seem to be accompanied by elevated expression of OXPHOS proteins. Presumably the levels of TCA cycle enzymes involved in substrate oxidation are also increased to match the increased electron transport capacity of the mitochondria. This is suggested by the elevated levels of the complex II subunit SDHB (complex II is also a direct participant in the TCA cycle) and could explain why only small or no changes are observed in studies of PD mitochondria that measure respiratory complex activity normalized against the activity of the TCA cycle enzyme, citrate synthase.

### Mitochondrial respiratory activity in iPD lymphoblasts is elevated regardless of patient age, disease duration or disease severity

The increased production of ROS that is a byproduct of elevated mitochondrial activity in iPD lymphoblasts could have several undesirable consequences for the cells. One is that it activates the cellular oxidative stress response and in a vicious feedback cycle then further stimulates mitochondrial biogenesis, activity and turnover. A second is that these reactive oxygen species, including hydrogen peroxide, hydroxyl radicals, superoxide and singlet oxygen, non-specifically damage key cellular macromolecules with which they come into contact, including proteins, DNA and membrane lipids. This could also contribute to a vicious feedback by eliciting greater turnover of both mitochondria and other cellular components with more ATP consumption and generation, consequential ROS production and macromolecular damage. Elevated ROS production in iPD cells is thus predicted to cause a gradual accumulation of damage to mitochondrial respiratory complexes, the proteins in closest proximity to the source of the ROS (Schapira, 2011; Schapira and Gegg, 2011; Keane et al., 2011; Exner et al., 2012; Dias et al., 2013). We therefore anticipated that as the disease progresses and becomes more severe, the initially hyperelevated mitochondrial respiratory function might decline. This could explain why respiratory complex activities are reduced in post-mortem iPD brains, even though they are not reproducibly lower in other types of cells derived from live individuals and, as we have shown, are in fact dramatically elevated in PD lymphoblasts.

**Fig. 5.**
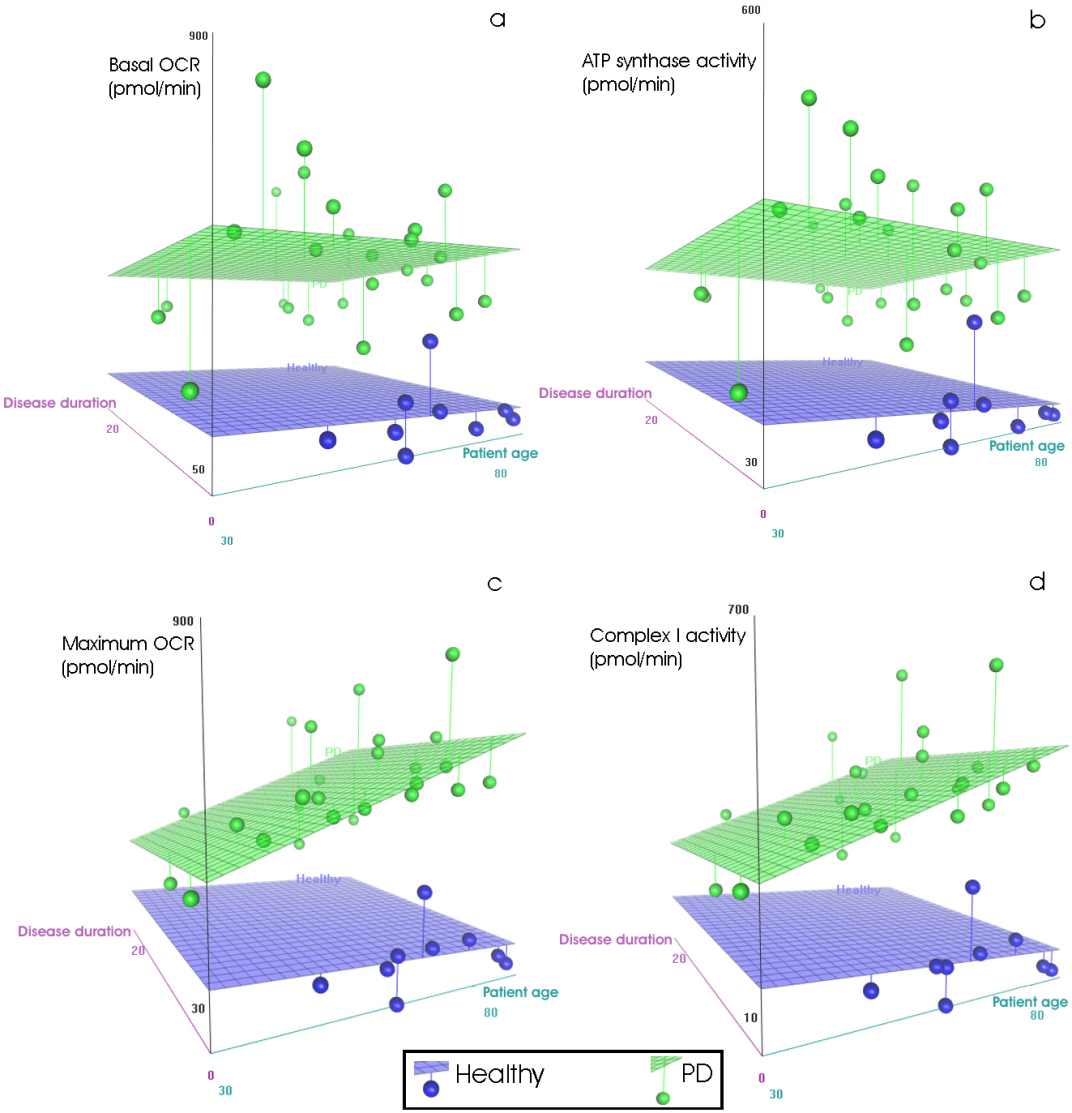
**Regression relationships between mitochondrial respiratory activity, patient age and disease duration.** The time since first diagnosis was recorded as a surrogate measure of disease duration. For plotting and analysis purposes, control participants were deemed to have disease durations of 0. Planar surfaces were fitted by least squares to show the relationships between patient age and disease duration and (A) basal OCR, (B) OCR attributable to mitochondrial ATP synthesis, (C) maximum uncoupled OCR and (D) maximum uncoupled OCR attributable to complex I. In multiple linear regressions incorporating successive removal of least significant coefficients and the use of dummy variables to distinguish between PD and control groups, the only significant regressions were those relating the maximum OCR (*P*=8.09×10^−4^, two-sided *t*-test) and complex I activity (*P*=2.09×10^−3^, two-sided *t*-test) to PD patient age (Fig. S2). Basal OCR, ATP synthesis, complex II activity, the proton leak and non-respiratory OCR were not significantly affected by patient age or disease duration (*P*>0.1, two-sided *t*-test).

To test if a decline in respiratory function during disease progression is evident in PD lymphoblasts, we examined whether there was a correlation between the various components of mitochondrial respiration, the age of the patient and the duration of clinical disease. We found that although the basal metabolic rate and the OCR attributable to ATP synthesis declined slightly both with patient age and with disease duration, neither of these effects was statistically significant in a multiple regression analysis (Fig. 5A,B; Fig. S2). Even in the oldest individuals with the longest times since diagnosis, mitochondrial ATP synthesis rates remained much higher than normal. When we examined if other components of lymphoblast respiration changed as individuals aged and disease progressed, we found that the only components of mitochondrial respiratory function that changed significantly were the maximum respiratory capacity and complex I activity after treatment with the uncoupling protonophore CCCP. Both increased with patient age but neither changed significantly with disease progression (time) since diagnosis (Fig. 5C,D). None of the other parameters of respiration in individuals with PD or control groups changed significantly with age or disease duration (Fig. S2). Likewise, the clinical measures of motor function and cognitive decline (see Materials and Methods) were not significantly correlated with any components of lymphoblast respiration considered here (exemplified in Fig. S3 with UPDRS motor scores, prorated IQ, basal respiration and ATP synthesis rates). Although these negative results have been obtained in a small number of subjects, we can infer that these relationships, even if found significant in a larger sample, would not be strong. Thus, the basal physiological state and metabolic need for ATP remained elevated in PD lymphoblasts regardless of the course of the disease and despite the high levels of ROS production.

### Elevated mitochondrial respiratory activity could form the basis of a blood test

**Fig. 6.**
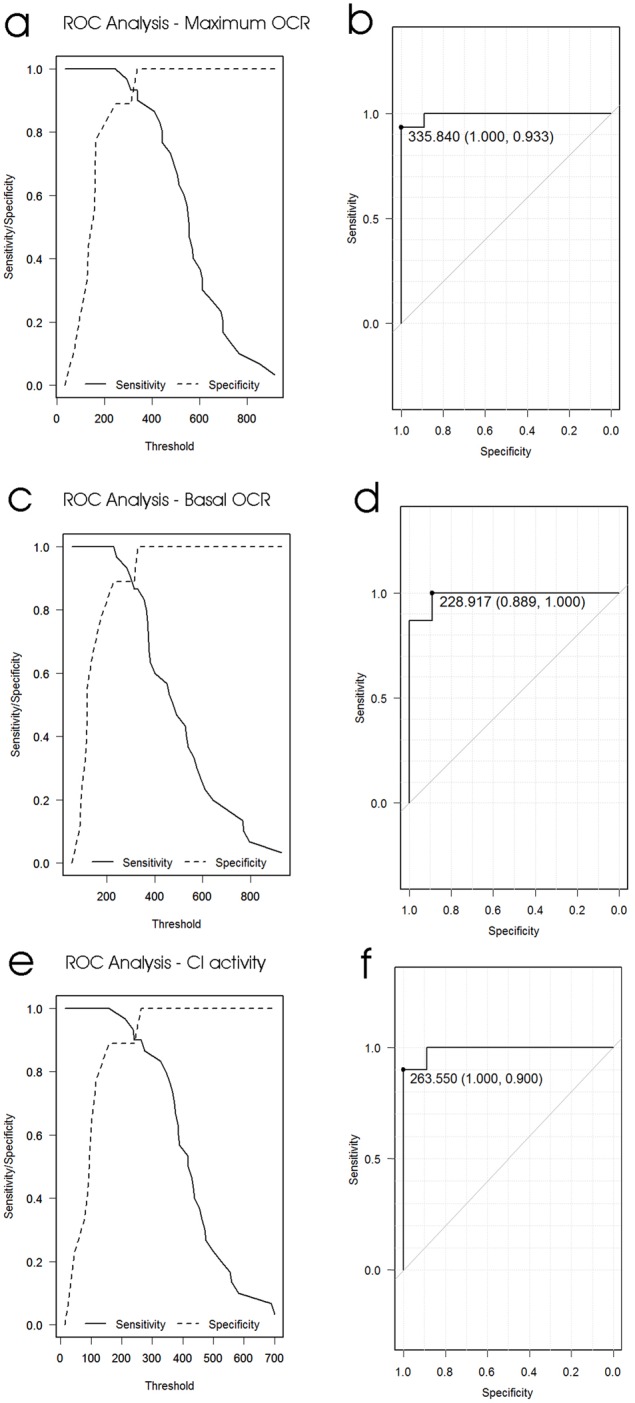
**ROC curve analysis of the use of respiration rates of lymphoblasts as diagnostic tests for PD.** The optimum test threshold was a maximum OCR of 335.84 pmol/min (A,B), a basal OCR of 228.92 pmol/min (C,D) or a complex I activity of 263.55 pmol/min (E,F). Lymphoblasts exceeding this threshold indicate PD with the indicated specificities (proportion of control individuals correctly excluded) and sensitivities (proportion of PD individuals correctly diagnosed). Areas under the curve (a measure of test reliability) were 0.9926 (95% confidence interval: 0.975-1.0, DeLong's test) for the maximum OCR, 0.985 (95% confidence interval: 0.953-1.0, DeLong's test) for the basal OCR and 0.989 (95% confidence interval: 0.964-1.0, DeLong's test) for complex I activity.

It is widely recognized that a sensitive and specific biomarker of Parkinson's disease is needed to assist diagnosis and prognosis as well as to test the efficacy of potential treatment regimes. Our results have confirmed that blood cells are entangled in PD pathology and have shown that several components of mitochondrial respiration in lymphoblasts could provide such biomarkers. Receiver operating characteristic (ROC) analysis of the use of the maximum uncoupled O_2_ consumption rate of PD lymphoblasts suggested that it could form the basis of a reliable test for PD (Fig. 6A,B; 100% specificity and 93.3% sensitivity, 99.3% reliability). Very similar and statistically indistinguishable results (by DeLong's test, Table S1) (DeLong et al., 1988) were found using basal respiration rates (Fig. 6C,D; *P*=0.48) and the respiratory activity of complex I (Fig. 6E,F; *P*=0.48). These results reflect the dramatic nature of the respiratory differences between lymphoblasts from the individuals with PD and control groups and need to be confirmed in larger studies. Furthermore, without wider studies encompassing other diseases, we cannot tell whether these respiratory differences are markers specifically of PD or more generally of neurodegeneration.

## DISCUSSION

Our results have confirmed that iPD blood cells are different from controls. In particular, the lymphocytes of individuals with iPD differ from controls not only in their global gene expression patterns (Soreq et al., 2013), but also in their metabolism when immortalized. Although these results need to be confirmed in a much larger study, they were dramatic – the rates of mitochondrial respiration, ATP synthesis and maximum, uncoupled O_2_ consumption rates in lymphoblasts from individuals with iPD were fourfold those of control cells.

Despite these large increases, the steady-state ATP levels of PD lymphoblasts were elevated only by about 75%, whereas the respiratory spare capacity was elevated only by about 10% of the basal OCR. These observations imply that the high rates of electron transport and mitochondrial ATP synthesis in PD lymphoblasts are matched by similarly increased rates of ATP consumption. Part of this additional energy consumption can be accounted for by increased rates of biogenesis of respiratory enzymes. It has been reported previously that accumulation of wild-type or a PD-causing mutant form of α-synuclein causes increased autophagic turnover even of normal mitochondria in neuronal cells (Choubey et al., 2011). This increased turnover of mitochondria is energetically expensive and supporting it would require the same steady-state mitochondrial mass to be more active in oxidative phosphorylation, as we observed. Another contributor to the elevated ATP consumption by PD lymphoblasts might well be the accelerated rate of growth of these cells that was previously reported (Esteras et al., 2015). It is of interest that whole-body resting respiration rates by individuals with iPD has also been known for many years to be elevated, but only in some individuals could this be attributed to the abnormal neuromuscular state (Levi et al., 1990).

The respiration, ATP synthesis and ROS production rates in iPD lymphoblasts remained elevated and unchanged in the face of patient ageing, ongoing disease and increased disease severity. The maximum OCR and complex I activity in uncoupled mitochondria increased modestly (as a percentage of the basal OCR) with patient age, but were unaffected by disease progression (time since diagnosis) and severity. How can these observations be reconciled with reports of impaired complex I activity in post-mortem PD brains? Unlike lymphoblasts and lymphocytes, brain neurons are long-lived, post-mitotic cells. Their longevity provides an opportunity for ROS-mediated damage to accumulate over time that does not pertain to the same extent to blood cells or their cultured derivatives. Even though PD leukocytes do exhibit elevated levels of the oxidative DNA base damage product 8-OH-2-deoxyguanosine (8-OHDG) (Chen et al., 2009), there is no evidence that oxidative damage to PD immune cells is sufficient to produce immunopathological outcomes. This stands in stark contrast to the affected brain regions of individuals with PD. It seems likely that the relatively rapid turnover of blood cells allows them to avoid the long-term adverse consequences of hyperactive mitochondria. PD lymphoblasts might thus represent cells from individuals with PD in which respiratory activity is dramatically and permanently elevated to an extent that is unaffected by disease progression. The implications of this are far-reaching.

First, the accumulated oxidative damage in the brains of individuals with PD could be caused cell-autonomously by initially hyperactive mitochondria in the neurons themselves, or by hyperactive lymphocytes and glial cells involved in the local inflammation that accompanies neurodegeneration in affected areas of PD brains (Perry, 2012; Mosley et al., 2012). Derived as they are from immune system cells, PD lymphoblasts might better represent activated inflammatory cells in the brain than other cell types. The permanent mitochondrial hyperactivity of these cells might be a major cause of ongoing neurodegeneration.

Second, it is clear that PD can sometimes originate from mitochondrial damage and impaired function, either from environmental toxins or from mutations that directly affect the mitochondria (Schapira and Gegg, 2011; Keane et al., 2011; Exner et al., 2012; Perier and Vila, 2012). Thus, although our patient cohort provided no examples of impaired mitochondrial respiration, forms of PD have been reported that do involve such loss of respiratory function. It seems probable that respirometric assays of lymphoblasts would allow a clear distinction to be made between what might be called primary mitochondrial and non-mitochondrial forms of PD. Such distinctions might be important for diagnosis, prognosis and determination of treatment options in the future, perhaps not only for PD but also for other neurodegenerative diseases in which mitochondrial dysfunction has been implicated, including Alzheimer's, Huntington's and motor neuron diseases.

Third, the respiratory competence of cultured lymphoblasts might reflect the physiological and molecular state of PD lymphocytes right from the onset of the disease, before clinical diagnosis, before treatment and before ROS-mediated cytopathological damage has begun. Gene expression patterns in PD lymphocytes differ from controls from early in the disease process (Soreq et al., 2013). In our patient cohort, the shortest time since diagnosis was about 2 years, whereas one of our patients was untreated. Lymphoblasts from both of these individuals exhibited full-blown elevation of respiratory activity. Elevated lymphoblast respiratory activity might thus be present in Parkinson's disease (or more generally in neurodegenerative disease) not only before any treatment has begun but also prior to clinical diagnosis and cellular disease pathology.

**Fig. 7.**
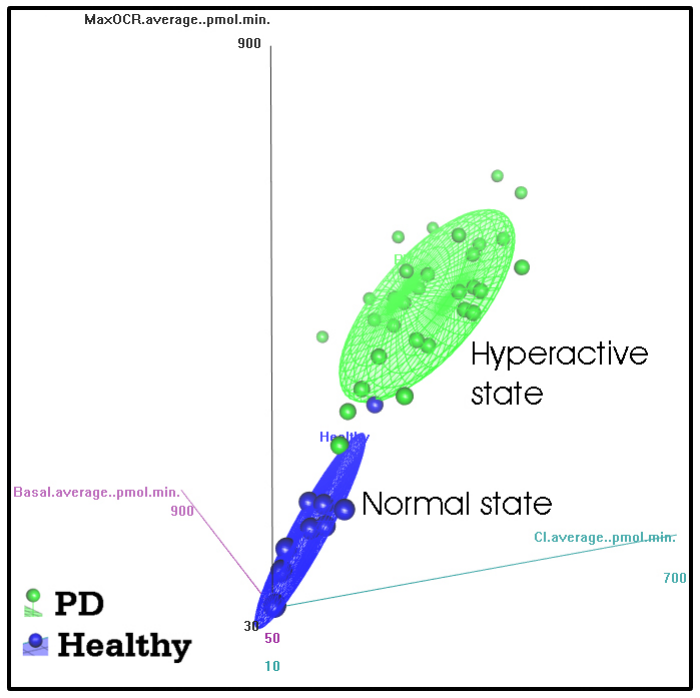
**PD and control lymphoblasts occupy distinct regions in multidimensional metabolic space.** The dramatic differences between PD and control lymphoblasts in respiratory and associated biochemical parameters can be visualized in the form of distinct regions of a multidimensional metabolic space. The figure illustrates this by depicting the median ellipsoidal surface fit to the results for three of the respiratory measures (OCR in pmol/min) that separated the two groups most clearly – complex I activity in the *x* axis, the maximum respiratory capacity in the *y* axis and the basal metabolic rate in the *z* axis. The existence of these two different metabolic states was confirmed in principal components analysis (Fig. S4), which accounts for and removes the effect of cross-correlations between variables.

Finally, the apparently permanent nature of the dramatic elevation in PD lymphoblast respiratory activity suggests that lymphoblasts can occupy two distinct quasi-stable steady states, a ‘normal’ and a ‘hyperactive’ state characterized by two different metabolic rates. Fig. 7 illustrates these two alternative metabolic states in a 3D-ellipsoidal median plot. The PD and control lymphoblasts occupy clearly separated volumes in a 3-D respiratory parameter space. The addition of the other respiratory and biochemical parameters would separate the PD and control lymphoblasts even further in what might be considered as multidimensional metabolic space. Our data show that lymphoblasts from PD and control individuals are found in these two states regardless of the progression of the disease. This being so, the beginning of the disease process could be initiated by an event that causes a switch from the normal to the hyperactive state. This could be an environmental insult or the outcome of some stochastic event, contingent upon the individual's genetic profile. Such bistable switches are characteristic of complex systems that involve multiple autoactivating and autoinhibiting homeostatic feedbacks. Perhaps the complex molecular pathways that have so far been implicated in PD are capable of such bistable switching and circumstances might exist that reverse the switch.

## MATERIALS AND METHODS

### Participant cohort

Data collection and analysis were conducted as part of a larger study on the role of the small CGG expansion FMR1 alleles in parkinsonism, supported by NIH, and approved by the Human Ethics Committees of La Trobe University, and of the Royal Melbourne Hospital in Melbourne, Australia. All participants were asked and gave informed consent for their involvement in the study. All individuals with PD and normal, healthy controls were white, male Caucasians aged from 28 to 85 and were of European (mainly northern European) origin, residing in Australia. Although the larger PD cohort included three individuals that were younger than the youngest of the controls, the age distributions of the two cohorts were not significantly different (Mann–Whitney *U*-test, *P*=0.96; Fig. S5). All the participants were recruited from the Movement Disorders clinic of the University of Melbourne and they were diagnosed by a movement disorders specialist (AE) as having idiopathic Parkinson's disease (iPD) according to the Parkinson's UK Brain Bank criteria (Hughes et al., 2002). Two individuals, aged 28 and 30 years, had atypical iPD in which early-onset parkinsonism was associated with epilepsy and psychiatric problems and were included because their cell biological and biochemical alterations were not different from those of the remaining iPD participants. One individual had unclassifiable parkinsonism and is under ongoing review. All patients were receiving treatment with levodopa and equivalents, with a daily dosage (LEDD) of 9.3 varying between individuals towards achieving an optimal effect. The treatment duration was, on average, one year less than disease duration. None of the individuals showed any evidence of dementia. Control participants were recruited separately and did not have any known neurological disorders or other conditions that might have been relevant to the study.

### Neurological and neuropsychological assessments

Standard neurological assessment and ratings consisting of the Unified Parkinson's Disease Rating Scale Part III–Motor (UPDRS–III) (Fahn and Elton, 1987) were conducted and scored by A.E. in subjects on their usual PD medications. All cognitive tests were administered by a trained neuropsychologist (E.H.). The Addenbrooke's Cognitive Examination–Revised (ACE–R) combines a number of subscales including attention and orientation, memory, fluency, language and visuospatial (Mioshi et al., 2006). The overall test score ranges from 0 to 100, with a cut-off score of <88 giving 94% sensitivity and 89% specificity for dementia (Mioshi et al., 2006). A number of subtests from the Wechsler Adult Intelligence Scale 3rd Edition (WAIS–III) included the Vocabulary subtest used to test basic word knowledge; Similarities and Matrix Reasoning used to test the aspects of executive functioning; and Digit Span used as the measures of working memory (Wechsler, 1997). The Matrix Reasoning and Vocabulary subtests were used to calculate a prorated IQ (Engelhart et al., 1999). The Symbol Digit Modalities Test (Smith, 1973) was employed as the measure of processing speed. This test was selected as it has an oral or verbal option, which allowed for assessment of processing speed without a motor component, which was important in this population.

### Isolation and maintenance of lymphoblastoid cell lines

Lymphoblastoid cell lines were created by EBV-mediated transformation of cells from the peripheral blood mononuclear cell (PBMC) layer at the interface of Ficoll-paque Plus (Sigma-Aldrich) gradients. In each case, an aliquot of 1×10^6^ PBMCs was transformed with EBV from the cell line B95.8 using cyclosporin A (Sigma-Aldrich) to reduce the innate immune response and rejection of the virus. Upon successful establishment, cell lines were expanded into larger cultures and stored at −80°C in multiple aliquots of 5×10^6^ cells in 500 µl Recovery Cell Culture Freezing Medium (Life Technologies, 12648010) before proceeding to experimentation. For experimental testing, aliquots were taken from storage and allowed to defrost at room temperature. The cells were transferred into 10 ml of fresh MEMAlpha medium supplemented with 10% foetal bovine serum (FBS) (Gibco) and 1% pen/strep (Gibco), then grown and maintained at 37°C in a humidified atmosphere with 5% CO_2_. All experiments were conducted within 10 passages of recovery from frozen storage. For the ROS assays, cell lines were cultured in RPMI (Gibco) supplemented with 10% FBS, 1% pen/strep and 1× Glutamax (Gibco) at 37°C in a humidified atmosphere of 5% CO_2_.

### AMPK activity assay

AMPK immunoprecipitation, AMPK activity assays and western blot analysis used lysates from confluent cell lines (∼25 ml) grown in T75 flasks, harvested, lysed in lysis buffer (50 mM Tris-HCl pH 7.4, 150 mM NaCl, 1 mM EDTA, 1 mM EGTA, 1% Triton X-100, 50 mM NaF, 5 mM sodium pyrophosphate) supplemented with phosphatase inhibitors then snap-frozen in liquid nitrogen. Thawed lysates were cleared by centrifugation at 10,000 ***g*** for 5 min. Supernatant total protein concentrations were determined with the Pierce™ BCA Protein Assay Kit (Thermo Fisher Scientific).

#### Antibody preparation and immunoprecipitation

Antibody preparation and immunoprecipitation were as described previously (Oakhill et al., 2010); 60 µl of ‘AMPK slurry’ was prepared from 1 mg of total protein lysate by immunoprecipitation using rabbit polyclonal anti-AMPKα1 antibody bound to equilibrated protein A-agarose beads. After overnight incubation at 4°C, the beads were recovered and washed four times by centrifugation before being resuspended in 60 µl wash buffer (50 mM HEPES pH 7.4, 150 mM NaCl, 10% glycerol, 0.1% Tween-20). The resulting ‘AMPK slurry’ provided 2×20 µl aliquots for AMPK assay and 20 µl for western blot analysis.

#### AMPK activity assay

AMPK activity was assayed over 10 min at 30°C by adding 20 µl of AMPK slurry to 15 µl buffer containing 100 µM SAMS synthetic peptide (NH_2_-HMRSAMSGLHLVKRR-COOH), 5 mM MgCl_2_, 50 mM HEPES pH 7.4, 0.1% Tween-20 and 1 mM DTT. Reactions were started by adding [γ-^32^P]-ATP (final concentration 200 μM) and stopped by spotting 21 µl onto P81 ion-exchange chromatography paper (Whatman, GE Healthcare). Liquid scintillation counting (Perkin Elmer) was used to measure the incorporation of ^32^P into the SAMS peptide. Duplicates were averaged and normalized against values from the same internal control cell line, C105, in each independent experiment.

#### Western blot of α-pT172 and AMPKα1 levels

The levels of α-pT172 phosphorylation in lymphoblast samples were detected by immunoblotting in the presence of a set of α-pT172-AMPK/AMPK internal standards ranging from 10-80%. The α-pT172 signal was detected using anti-α-pT172 antibody (1:1000; Cell Signaling Technologies, 2535S), followed by incubation with secondary anti-rabbit IR680 (1:5000; LI-COR Biosciences, 925-68071). Total AMPKα1 signal was detected using AMPKα1 antibodies as described previously (Oakhill et al., 2010) fluorescently labelled with IR800. Immunoblots were visualised on an Odyssey infrared imaging system (LI-COR Biosciences).

### Mitochondrial membrane potential and mitochondrial mass

Two mitochondrial dyes, MitoTracker Green FM and MitoTracker Red CMXRos (Thermo Fisher Scientific), were used to estimate mitochondrial mass and membrane potential (Pendergrass et al., 2004; Cottet-Rousselle et al., 2011; Martins et al., 2015; Henzi and Schwaller, 2015; Barbato et al., 2015). Fluorescence microscopy confirmed that both dyes stained the mitochondria with very little background staining of the cytosol (data not shown). Three aliquots of 1×10^6^ harvested cells were suspended in 1 ml Dulbecco's phosphate buffered saline (PBS) (Sigma-Aldrich) for 1 h at 37°C under 5% CO_2_, then transferred to: (1) fresh PBS, (2) fresh PBS containing 200 nM MitoTracker Red CMXRos or (3) fresh PBS containing 200 nM MitoTracker Green FM. After incubation in the dark at 37°C and 5% CO_2_ for 1 h, each aliquot of cells was transferred to 2 ml fresh PBS for fluorescence measurements using a Modulus Fluorometer (Turner Biosystems, Sunnyvale, CA). The Green Module (excitation 525 nm, emission 580-640 nm) was used for MitoTracker Red CMXRos and the Blue Module (excitation 460 nm, emission 515-570 nm) for Mitotracker Green. The unstained aliquot of cells in PBS was used in each case to measure background fluorescence. The background-subtracted green fluorescence measured mitochondrial mass and the ratio of the red and green background-subtracted fluorescences measured the membrane potential. Measurements were made in duplicate for each cell line, averaged and normalized within every experiment to the values for the same arbitrarily selected control cell line (C105).

### Respirometry

The Seahorse XF^e^24 Extracellular Flux Analyzer (Seahorse Bioscience) was used to measure the oxygen consumption rate (OCR), a measure of mitochondrial respiration in real-time in live intact lymphoblastoid cells. Prior to inoculating cells, a 24-well PS cell culture plate was pre-coated with 4.5 µl Matrigel growth factor-reduced (GFR) basement membrane matrix, Phenol-Red free, LDEV-free, 10 ml (Corning, 356231), diluted 1:2 in XF assay medium (unbuffered DMEM supplemented with 2.5 mM glucose and 1 mM sodium pyruvate) and then allowed to dry completely. One hour prior to the assay 8×10^5^ cells in XF assay medium were inoculated into the Matrigel-coated wells. Each cell line was inoculated into four replicate wells. Several measures of mitochondrial respiration, including basal respiration, ATP-linked respiration, proton leak respiration, maximum respiration rate and spare respiratory capacity, were derived from oxygen consumption rates before and after the sequential addition of pharmacological agents (1 µM oligomycin, 1 µM CCCP, 1 µM rotenone, 5 µM antimycin A) to the respiring cells. Between successive additions, the O_2_ consumption rate was measured and averaged over three measurement cycles (time points), each cycle consisting of a mix step of 3 min, a wait of 2 min and a measurement time of 3 min.

### ATP assay

Steady­-state ATP levels were measured as per the manufacturer's instructions using the ATP Determination Kit (Molecular Probes) and 100 µl of clarified cell lysate from 5×10^5^ cells, diluted with 900 µl 28 mM tricine buffer (pH 8.25). Luciferase-mediated ATP-driven luminescences were measured in duplicate aliquots in the Modulus Fluorometer with the chemiluminescence module (Turner Biosystems). Average background-subtracted luminescences were used to determine ATP concentrations from a standard curve constructed using tenfold serial dilutions of the ATP standard (100 nM-1 µM). In each experiment, mean ATP levels (nmol per 10^6^ cells) were normalized against the values obtained for a control cell line (C101) within the same experiment.

### Mitochondrial DNA copy number

Total DNA was extracted from ∼5×10^6^ cells using DNAzol (Molecular Research Center, Inc.) according to the manufacturer's instructions. To measure mitochondrial DNA (mtDNA) copy number, semi-quantitative real-time PCR was employed using the iQ5 real-time PCR detection system and the SensiFAST SYBR & Fluorescein One-Step Kit (Bioline) for amplicon detection. A fragment of each of the mitochondrial ND1 and ND4 genes was amplified using the primers ND1F (5′-cacccaagaacagggtttgt-3′) and ND1R (5′-tggccatgggattgttgttaa-3′), and MTND4F (5′-caaccttttcctccgacccc-3′) and MTND4R (5′-ctggataagtggcgttggct-3′). For internal loading controls, fragments of the nuclear-encoded mitochondrial gene β2-microglobulin and the 18SrRNA subunit were amplified, using the primers B2-MGF (5′-cactaggaccttctctgagc-3′) and B2-MGR (5′-ctacagcttgggaattcctgc-3′) for β2-microglobulin and 18SrRNAF (5′-tagagggacaagtggcgttc-3′) and 18SrRNAR (5′-cgctgagccatgcagtgt-3′) for 18S rRNA. The threshold cycle numbers obtained for the ND1 and ND4 genes were subtracted from those for the β2-microglobulin and 18S rRNA genes to give normalized measures for the relative mitochondrial genome copy number.

### Mitochondrial gene expression

RNA was extracted from ∼5×10^6^ cells using TRIsure (Bioline), treated with RQ1 DNase (Promega) according to the manufacturer's instructions. To measure gene expression, semi-quantitative reverse transcription (RT)-PCR was used with the SensiFAST SYBR & Fluorescein One-Step Kit. Fragments of the mitochondrial ND1 and ND4 cDNA were amplified using the primers ND1F and ND1R, and MTND4F and MTND4R, respectively, whereas a fragment of the 18SrRNA subunit rRNA was amplified as the internal loading control using the primers 18SrRNAF and 18SrRNAR. The threshold cycle numbers obtained for the ND1 and ND4 genes were subtracted from those for the 18S rRNA gene to give an estimate of the relative level of mitochondrial gene expression. In a small subset of experiments the primers B2-MGF and B2-MGR were also used to amplify β2-microglobulin mRNA as an internal mRNA loading control and produced similar outcomes.

### Semi-quantitative western blotting of mitochondrial OXPHOS protein levels

Cells were lysed in SDS loading buffer (0.125 M Tris hydrochloride, 10% glycerol, 4% SDS, 4 M urea, 10% mercaptoethanol and 0.0001% Bromophenol Blue) with a protease inhibitor cocktail (cOmplete EDTA-free, Roche). A small aliquot of each briefly sonicated sample was analysed for total protein concentration using a Qubit Protein Assay Kit and Qubit 2.0 Fluorometer (Thermo Fisher Scientific) according to the manufacturer's instructions.

The samples were then heated to 85°C for 5 min and 300 µg of total protein was loaded into each well in 12% SDS polyacrylamide gels. After electrophoresis, proteins were transferred onto PVDF membranes (Amersham Hybond-P, GE Healthcare) using a Trans Blot Turbo Blotting apparatus (Bio-Rad) for 30 min at 25 V, 1.0 A, blocked for 1 h with blocking buffer (5% skim milk, TBS) and incubated overnight with primary antibodies (Total OXPHOS human WB antibody cocktail, Abcam, ab110411) diluted 1:250 in blocking buffer. This cocktail is directed against five OXPHOS proteins, of which only SDHB (29 kD) and ATP5A (54 kD) gave clear fluorescence signals using Alexa Fluor 488-labelled secondary antibody. An anti-alpha-tubulin antibody (1:1000; Abcam, ab52866) or an anti-β-actin antibody (1:3000; Santa Cruz Biotechnologies, sc-47778) was used as an internal loading control in combination with an Alexa Fluor 647-labelled secondary antibody. The secondary antibodies (diluted 1:1000 in TBS) were A11008 (Alexa Fluor 488 goat anti-rabbit IgG) and A21235 (Alexa Fluor 647 goat anti-mouse IgG) both from Thermo Fisher Scientific. Following incubation with antibodies the membranes were washed three times with TBS buffer containing 0.5% Tween 20, scanned with a Typhoon FLA7000 and analysed using the Image Quant TL 1D v 8.1 software (GE Healthcare Life Sciences).

### Reactive oxygen species levels

Intracellular ROS were detected in live cells using the Fluorometric Intracellular ROS kit (Sigma-Aldrich). Cells were harvested and resuspended in Dulbecco's phosphate buffered saline (Sigma) at 5×10^5^ cells/ml. To 500 µl of cells in microcentrifuge tubes, 600 µl of Master Reaction Mix was added from the Fluorometric Intracellular ROS kit (Sigma-Aldrich) after which the cells were incubated in darkness under 5% CO_2_ at 37°C for 1 h. The fluorescence (λ_ex_=520 nm/λ_em_=605 nm) was measured in a Modulus Fluorometer (Turner Biosystems) using the Green Module. An arbitrarily selected control cell line (C105) was used in every experiment as an internal, normalization control for between-experiment variation. The fluorescence is proportional to the amount of ROS present. In separate control experiments the fluorescence was shown to be quenched dramatically by addition of 100 mM mannitol, a colourless ROS trap that works by reacting with hydroxyl radicals (data not shown).

### Statistical analysis

All statistical and graphical analysis was conducted using R (R Core Team, 2013) and the R packages RCommander (Fox, 2005) and REzy (Kanda, 2013). Unless otherwise specified, two-sample tests used the Welch *t*-test. The significance of individual coefficients in multiple regression analysis was tested using *t-*tests. ROC (receiver operating characteristic) analysis was applied to determine the efficacy of respiratory parameters for diagnostic purposes and was as described by Fawcett (2006) and Zou et al. (2007).
